# 

**Published:** 1914-12-12

**Authors:** 


### CONTENTS.

PAGE

**Taxation and the Voluntary Hospitals ... ...** 231

**Women Doctors and the Present Crisis ...** 232

**Hospital and Institutional News ... ... ...** 233

A House Governor's Election—The New House Governor of the Birmingham General Hospital —A Hospital that Ran Away—Another Secretary on Active Service—Flashlights in Our War News Paragraphs—Privates Well Treated in German Hospitals—Boots for the Belgian Wounded—"The Glamorgan and Monmouth Hospital for the French Army"—Homes for Incurables and the War—Honorary Staffs and Hospital Boards—Medical Men as Eager Committeemen—Secretary and House Surgeon to Dispenser and Secretary—£10,000 for the Walsall Hospital—The New Master in Lunacy —The Spirit of the R.A.M.C.—Medical Men as Prisoners of War—A Devonport Hospital and Municipal Aid—The War and Country Practice—The Task of the British Red Cross Society—The Central Council for District Nursing—The Success of a Central Drug Stores—The Question of Christmas Festivities —The London Ambulance Service—Effect of the War on the Drug Supply—The Manufacture of Synthetic Drugs—The League of Mercy Annual Meeting—The Sardonic Porter— This Week's Drug Market.

**Mental Stress Associated with Shock ... ...** 239

Some Obscure Results of Violence in Peace and War.

**Abdominal Cæsarean Section for Eclampsia ...** 240

**A Hospital for Soldiers Suffering from Shock** 241

Dr. Edwin Ash's Reply to Lord Knutsford.

**Round the World ... ... ... ... ... ...** 242

Fellow-Workers and the Roll of Honour.

**Reports on Hospitals of the United Kingdom—** 243

By SIR HENRY BURDETT, K.C.B., K.C.V.O.

The Halifax Union Poor-Law Hospital.

[*See over.*]

### CONTENTS—(*continued*).

PAGE
**Hospital Economics** ... ... ... ... ... 245
King Edward's Hospital Fund for London Statistics.
**Personalia** ... ... ... ... ... ... ... 246
**Hospitals and the War** ... ... ... ... 247
**Work for the Wounded of the Allied Armies**
Cambridge: More Wounded Arrive—Cardiff: Weather in France—Leicester: 5th Northern General Hospital—Wakefield: Clayton Hospital—Bristol—Doncaster: Mansion House for Refugees—Sheffield: A Soldier on Doctors Under Fire.
**The Book Market** ... ... ... ... ... ... 248
**The Editor's Letter-Box** ... ... ... ... 249
The Medical Research Committee and the War.
Vacant Appointments: Canvassing or Non-Canvassing for Elections.

PAGE
"The War's Effect on a London Eye Hospital."
A Suggested New Flag for Hospitals.
**The Institutional Library** ... ... ... ... 250
French Practice of Urgent Surgery.
A Practical Book for Residents.
**Medical Appointments** ... ... ... ... 252
**The Institutional Worker** ... ... (Suppl.) 1
Answer to November Question.
Sterile Water for Operation Theatres.
An Installation at a North-country Infirmary.
Accounting at a Nursing Home.
Questions for December.
Questions Invited.
The Sick and in Need.
War Matters.
Employment and Training.
Practical Matters.

